# Association between p-tau181 in cerebrospinal fluid and shunt response in patients with normal-pressure hydrocephalus

**DOI:** 10.1007/s10072-026-09357-x

**Published:** 2026-09-07

**Authors:** Amon Bergner, Christian Sandøe Musaeus, Anja Hviid Simonsen, Tina Nørgaard Munch, Jonathan Frederik Carlsen, Steen Gregers Hasselbalch

**Affiliations:** 1 Danish Dementia Research Centre, Section 8008, Department of Neurology, Copenhagen University Hospital, Inge Lehmanns Vej 8, DK-2100, Copenhagen, Rigshospitalet Denmark; 2 Department of Clinical Medicine, Faculty of Health and Sciences, University of Copenhagen, Copenhagen, Denmark; 3 Department of Neurosurgery, Copenhagen University Hospital, Rigshospitalet, Copenhagen, Denmark; 4 Department of Radiology, Copenhagen University Hospital, Rigshospitalet, Copenhagen, Denmark; 5 Department of Congenital Disorders, Statens Serum Institut, Copenhagen, Denmark

**Keywords:** Normal-pressure hydrocephalus, Cerebrospinal fluid, P-tau181, Shunt response

## Abstract

**Background:**

Idiopathic normal pressure hydrocephalus (iNPH) is a treatable condition, but predicting shunt response remains challenging. Cerebrospinal fluid (CSF) biomarkers, including phosphorylated tau (p-tau181), have been suggested as potential predictors, although existing evidence is inconsistent.

**Methods:**

In this retrospective single-center study, we included 230 patients with probable or possible iNPH who underwent shunt surgery and had available CSF biomarker data for Amyloid beta42, total tau and phosphorylated tau 181. Patients were divided into two cohorts based on assay method (Innotest^®^ and Elecsys^®^). The primary outcome was improvement in gait score. Secondary outcomes included cognition, continence, and overall clinical response. Predictive performance of CSF p-tau181 was evaluated using logistic regression and receiver operating characteristic (ROC) analyses.

**Results:**

Mean CSF p-tau181 levels did not differ significantly between responders and non-responders in either cohort. In multivariable logistic regression analyses adjusted for age and sex, CSF p-tau181 was not associated with shunt response (Innotest^®^: OR 0.997 (0.972–1.024), *p* = 0.815; Elecsys^®^: OR 1.091 (0.910–1.419), *p* = 0.429). Findings were consistent across two CSF p-tau181 assay platforms, although the Elecsys^®^ cohort was underpowered for calculating AUC with only 14 non-responders. Similar results were observed across secondary outcomes and after exclusion of patients with high CSF p-tau181 levels to reduce potential confounding by concomitant Alzheimer’s disease pathology.

**Conclusion:**

CSF p-tau181 does not demonstrate clinically meaningful predictive value for shunt response in patients with iNPH and cannot alone guide patient selection for shunt surgery. Future research should focus on multimodal approaches integrating clinical, radiological, and biochemical markers.

**Supplementary Information:**

The online version contains supplementary material available at https://doi.org/10.1007/s10072-026-09357-x.

## Introduction

Idiopathic normal pressure hydrocephalus (iNPH) is a disorder characterized by ventricular enlargement despite normal intracranial pressure. The etiology and pathophysiology remain uncertain but appear to involve impaired CSF resorption and glymphatic system dysfunction [1–3]. Patients typically present with the classic clinical triad of gait disturbance, cognitive impairment, and urinary urgency or incontinence [4, 5]. Typical radiological features include ventriculomegaly, narrowed callosal angle, widening of the Sylvian fissures and sulcal crowding at the vertex [6, 7].

Currently, CSF shunting, most commonly by ventriculoperitoneal shunt implantation, is the only effective treatment and leads to a favorable outcome in approximately 80% of patients [8–11].

As complications (e.g. overdrainage, infection, subdural hematoma) are frequent [12], it is essential to identify patients eligible for surgery and predict whether an individual patient will benefit from shunt placement. Typical MRI findings in combination with symptoms and supplementary tests, such as tap test and lumbar infusion tests, are commonly used to predict shunt response [5, 13–16] and show moderate positive and poor negative predictive value. Thus, accurately identifying responders remains challenging.

One method of identifying responders could be cerebrospinal fluid biomarkers, but patients with iNPH often have coexisting conditions with overlapping symptomatology, such as Alzheimer’s disease (AD) and cerebral small vessel disease [17]. So far, studies have focused on CSF biomarkers specific for AD pathology – such as amyloid beta 40 and 42 (Aβ42), phosphorylated tau (p-tau) and total tau (t-tau) – to understand their role in predicting shunt outcome. Tau is a microtubule-associated protein, and hyperphosphorylation of tau leads to axonal destabilization and ultimately promotes neurodegeneration [18]. On a group level, iNPH patients show reduced levels of Aβ42 similar to AD but normal levels or even below-normal levels of p-tau phosphorylated at Threonine 181 (p-tau181), compared to healthy controls [19–21]. Interestingly, levels of p-tau181 and t-tau may even be lower than in healthy controls, and combined with low Aß42, this pattern remains incompletely understood but has been interpreted as reflecting impaired protein clearance from the brain [22].

However, the typical pattern of abnormal biomarkers seen in CSF in AD – low Aβ42 and high p-tau181 – is seen in some patients with iNPH, probably reflecting concomitant AD pathology [23]. Studies investigating the predictive power of AD biomarkers in CSF have shown conflicting results, although some point to p-tau181 in CSF as a potential predictor of shunt response [24–38]. A recent study by Grønning et al. reported no association between CSF p-tau181 and shunt response [38]. However, studies addressing this issue remain scarce and most are based on small patient cohorts. Therefore, we aimed to further evaluate the association between CSF p-tau181 and shunt outcomes in iNPH in one of the largest cohorts reported to date [24–30]. The primary outcome was prediction of improvement in gait with the secondary outcomes being prediction of improvement in cognition, continence, and overall clinical response. Given that low CSF p-tau181 levels are characteristic of iNPH, we hypothesized that lower CSF p-tau181 concentrations would be associated with a positive effect of shunt treatment.

## Methods

### Study design and setting

This is a retrospective single-center study conducted at the Memory Clinic in collaboration with the Department of Neurosurgery, Rigshospitalet, Copenhagen, including patients diagnosed with probable or possible iNPH [39] between 2013 and 2025.

According to Danish legislation (VEJ nr 11052 af 02/07/1999), the need for explicit written consent was not required since the study was approved as a quality assurance study by the director of the Neuroscience Center, Rigshospitalet (see approval document in supplementary material). Quality assurance studies do not need approval from the ethical committee system in Denmark. However, all patients gave informed consent for their data to be stored and used for future studies which was approved by the Danish Data Protection Agency (Journal nr 2007–58-0015).

### Clinical evaluation

Diagnostic work-up of patients suspected of iNPH included patient anamnesis, physical and neurological examination, blood screening tests, structural brain imaging: magnetic resonance imaging (MRI) or computed tomography (CT), and either a lumbar tap-test with withdrawal of 40 mL (TT), a lumbar infusion test (LIT), or both. Diagnosis was reached by consensus at a multidisciplinary team conference with the Department of Neurosurgery based on international diagnostic criteria [39]. Positive test results (TT or LIT) supported the decision to proceed with shunt implantation, whereas negative results did not preclude the insertion of a shunt.

Details of the clinical evaluation have been described previously by Hasselbalch et al. [10]. Briefly, the diagnostic work-up included a standardized neurological examination with assessment of gait using a 10-m walk test scored according to Hellström et al. [40], cognitive function assessed by the Mini-Mental State Examination (MMSE) and the Addenbrooke’s Cognitive Examination (ACE). Urinary continence was evaluated through interviews with patients and/or caregivers and scored according to Hellström et al. [40]. Structural brain imaging was performed using MRI or CT in patients with contraindications to MRI.

### CSF biomarker analysis

CSF was obtained through preoperative lumbar puncture as part of the supplementary TT or LIT and routinely analyzed for t-tau, p-tau181 and Aβ42.

CSF Aβ42 concentration was measured using sandwich ELISA (INNOTEST^®^ β-AMYLOID_(1–42)_ Fujirebio), and CSF p-tau181 levels were determined using ELISA INNOTEST^®^ PHOSPHO-TAU_(181P)_ before July 1, 2022. CSF collected after July 1, 2022, was analyzed with the Elecsys^®^ sandwich electrochemiluminescence-immunoassay (Roche, Cobas 8000) [41, 42]. The two cohorts were mutually exclusive, as each patient’s CSF sample was analyzed using the assay platform that was in clinical use at the time of sample collection.

### Study population

A total of 230 patients met the inclusion criteria: 1) diagnosis of iNPH, 2) availability of CSF biomarkers (t-tau, p-tau181, Aβ42), 3) underwent shunt surgery, and 4) documented clinical follow-up of at least 2 months.

### Shunt placement and shunt response evaluation

Surgical placement of a ventriculo-peritoneal shunt was performed according to standard procedures at the Department of Neurosurgery at Rigshospitalet. Either a flow-regulated, fixed pressure valve or a differential pressure valve with antisiphon was used, as preferred by the treating neurosurgeon. Shunt response was evaluated at follow-up, at least 2 months after shunt surgery or, in cases of complications, 2 months after resolution. Outcomes for cognition, continence and gait were assessed using the same methods as preoperatively.

Changes in gait and continence were assessed using the scale by Hellström et al. [40]. Outcomes were categorized as worsening (increase in score ≥ 1), no change, moderate improvement (decrease in score by 1) or substantial improvement (decrease in score ≥ 2).

Cognitive outcome was evaluated using the Mini-Mental State Examination (MMSE) and categorized as worsening (decrease ≥ 2 points), no change (−1 to + 1 points), moderate improvement (+ 2 points), or substantial improvement (≥ + 3 points).

For overall shunt response score, outcomes for gait, cognition and continence were converted to a scale ranging for −1 to 2 (worsening −1, no change 0, moderate improvement 1, substantial improvement 2) and summed to yield a composite score ranging from −3 to 6.

For this study, we chose the primary outcome as motor response following shunt surgery, defined as improvement in gait score at follow-up as gait disturbance is the most treatment-responsive symptom in iNPH [43]. In statistical analyses, shunt responders were defined as patients with a gait response score decrease from baseline to follow up ≥ 1. Secondary analyses were performed using changes in cognition and continence and overall clinical response as outcomes using the scoring system described above.

### Statistical analyses

Statistics were performed using the statistical software R (version 4.5.2).

Group differences between gait responders and non-responders were evaluated using Student’s t-test or Welch’s t-test for continuous data, depending on variance equality. If the data were not normally distributed, we log-transformed the data. In cases where log-transformation was insufficient or the data were not continuous, we used Wilcoxon Rank Sum Test. We used chi-square test or Fisher’s exact test for categorical variables based on whether the number of subjects in each group was above 5.

The discriminative ability of CSF p-tau181 to predict shunt response was evaluated using ROC analysis, including calculation of the area under the curve (AUC). In addition, we performed multivariable logistic regression models adjusted for age and sex.

As AD pathology is prevalent in patients with iNPH [35, 36], and may alter shunt response, we sought to estimate the influence of AD co-pathology on the association between p-tau181 levels and shunt response. Higher p-tau181 levels were considered to increase the likelihood of AD co-pathology and therefore, the ROC analysis was repeated after excluding patients with the highest 10%, 20% and 30% p-tau181 levels. This analysis was intended to assess whether markedly elevated p-tau181 values potentially related to concomitant AD pathology influenced the primary findings, rather than identifying patients with AD.

As p-tau181 was analyzed using different assay methods before June 30, 2022 (Innotest^®^) and from July 1, 2022, onward (Elecsys^®^), the total cohort was divided accordingly. All analyses were performed identically in both assay cohorts.

## Results

A total of 230 patients meeting the inclusion criteria were divided into an Innotest^®^ cohort (*n* = 168) and Elecsys^®^ cohort (*n* = 62). In the Innotest^®^ cohort, 119 were defined as shunt responders (70.8%), whereas 48 patients were classified as shunt responders in the Elecsys^®^ cohort (77.4%). Baseline characteristics are presented in Tables 1a and 1b.

**Table 1a:** Innotest^®^

**Variable**	**Total** (***n* = 168)**	**Responder** (***n* = 119)**	**Non-responder** (***n* = 49)**	***p*-value**
Age (mean ± SD)	74.3 ± 6.2	74.6 ± 5.7	73.8 ± 7.3	0.496
Male (n, %)	104 (61.9%)	78 (65.5%)	26 (53.1%)	0.180
Preoperative MMSE (mean ± SD)	25.7 ± 3.9	26 ± 3.4	24.8 ± 4.9	0.109
Preoperative gait score (mean ± SD)	3.8 ± 1.6	4 ± 1.6	3.4 ± 1.5	**0.012**
Preoperative continence score (mean ± SD)	2.9 ± 1.2	2.9 ± 1.2	2.7 ± 1.4	0.289
Preoperative Tap-test (% mean ± SD)	−21.5 ± 24.3	−21.2 ± 26.6	−22.1 ± 18.1	0.528
Comorbidities (n, %)				
Neurological	70 (41.7%)	46 (38.7%)	24 (49%)	0.288
Musculoskeletal	63 (37.5%)	42 (35.3%)	21 (42.9%)	0.456
Urogenital	28 (16.7%)	19 (16%)	9 (18.4%)	0.879
Time to follow-up, days (mean ± SD)	242.6 ± 107.1	240.2 ± 102.6	248.3 ± 118.2	0.755
Preoperative levels of CSF biomarkers (mean ± SD)				
Total Tau (ng/L)	183.1 ± 107.4	181.3 ± 111.8	187.6 ± 96.8	0.508
pTau181 (ng/L)	30.7 ± 13.4	30.7 ± 13.8	30.8 ± 12.4	0.829
Aβ42 (ng/L)	692.8 ± 228.8	694.9 ± 228.1	687.6 ± 232.6	0.852
pTau181/Aβ42 ratio	0.052 ± 0.042	0.052 ± 0.045	0.052 ± 0.035	0.768

Table 1b Elecsys^®^				
**Variable**	**Total (*n* = 62)**	**Responder (*n* = 48)**	**Non-responder (*n* = 14)**	***p*-value**
Age (mean ± SD)	74.7 ± 5	74.8 ± 5.2	74.4 ± 4.2	0.793
Male (n, %)	35 (56.5%)	27 (56.2%)	8 (57.1%)	1
Preoperative MMSE (mean ± SD)	26.5 ± 3.3	26.3 ± 3.5	27.4 ± 2.5	0.289
Preoperative gait score (mean ± SD)	3.9 ± 1.2	4.1 ± 1.3	3.3 ± 0.7	**0.043**
Preoperative continence score (mean ± SD)	2.8 ± 1.4	2.9 ± 1.4	2.2 ± 1.3	0.095
Preoperative Tap-test (% mean ± SD)	−19.2 ± 12.5	−19.4 ± 13.1	−18.5 ± 10.5	0.766
Comorbidities (n, %)				
Neurological	26 (41.9%)	18 (37.5%)	8 (57.1%)	0.316
Musculoskeletal	28 (45.2%)	23 (47.9%)	5 (35.7%)	0.616
Urogenital	9 (14.5%)	7 (14.6%)	2 (14.3%)	1
Time to follow-up, days (mean ± SD)	299.2 ± 119.3	317.6 ± 127.6	236.3 ± 49.2	**0.003**
Preoperative levels of CSF biomarkers (mean ± SD)				
Total Tau (ng/L)	123.5 ± 32.6	123.8 ± 32.9	122.7 ± 32.8	0.986
pTau181 (ng/L)	10.9 ± 3.7	11.1 ± 3.9	10.1 ± 3.3	0.365
Aβ42 (ng/L)	621.6 ± 251	603.8 ± 256.5	682.7 ± 229.2	0.305
pTau181/Aβ42 ratio	0.02 ± 0.01	0.021 ± 0.01	0.017 ± 0.009	0.104

Across cohorts, time to follow-up ranged from 70 to 735 days after surgery with a mean follow-up time of 257.8 ± 113.1 days.

In both cohorts, responders had a significantly higher mean preoperative gait score (worse gait) compared to non-responders (Innotest^®^ (mean ± SD): 4.0 ± 1.6 vs. 3.4 ± 1.5, *p* = 0.012; Elecsys^®^: 4.1 ± 1.3 vs. 3.3 ± 0.7, *p* = 0.043). In the Elecsys^®^ cohort, responders also had a significantly longer time to follow-up compared to non-responders (317.6 ± 127.6 vs. 236.3 ± 49.2, *p* = 0.003), whereas no significant difference in time to follow-up was observed in the Innotest^®^ cohort.

When evaluating clinical outcomes (Tables 2a and 2b), the only significant difference – apart from gait improvement, which defined responder status – was improvement in urinary incontinence among responders in the Innotest^®^ cohort. In the same cohort, a trend toward improved cognition was also observed among responders.

**Table 2a:** Innotest^®^

**Variable**	**Total (*n* = 168)**	**Responders (*n* = 119)**	**Non-responders (*n* = 49)**	***p*-value**
Effect on gait				
Total positive effect (n, %)	119 (70.8%)	119 (100%)	0 (0%)	-
Substantial (n, %)	60 (35.7%)	60 (50.4%)	0 (0%)	-
Moderate (n, %)	59 (35.1%)	59 (49.6%)	0 (0%)	-
None (n, %)	43 (25.6%)	0 (0%)	43 (87.8%)	-
Worsening (n, %)	6 (3.6%)	0 (0%)	6 (12.2%)	-
Effect on urinary incontinence				
Total positive effect (n, %)	74 (44%)	62 (52.1%)	12 (24.5%)	0.002
Substantial (n, %)	26 (15.5%)	23 (19.3%)	3 (6.1%)	-
Moderate (n, %)	48 (28.6%)	39 (32.8%)	9 (18.4%)	-
None (n, %)	84 (50%)	56 (47.1%)	28 (57.1%)	-
Worsening (n, %)	10 (6%)	1 (0.8%)	9 (18.4%)	-
Effect on cognition				
Total positive effect (n, %)	59 (35.1%)	47 (39.5%)	12 (24.5%)	0.094
Substantial (n, %)	35 (20.8%)	27 (22.7%)	8 (16.3%)	-
Moderate (n, %)	24 (14.3%)	20 (16.8%)	4 (8.2%)	-
None (n, %)	98 (58.3%)	66 (55.5%)	32 (65.3%)	-
Worsening (n, %)	11 (6.5%)	6 (5%)	5 (10.2%)	-

Table 2b Elecsys^®^				
**Variable**	**Total (*n* = 62)**	**Responders (*n* = 48)**	**Non-responders (*n* = 14)**	***p*-value**
Effect on gait				
Total positive effect (n, %)	48 (77.4%)	48 (100%)	0 (0%)	-
Substantial (n, %)	18 (29%)	18 (37.5%)	0 (0%)	-
Moderate (n, %)	30 (48.4%)	30 (62.5%)	0 (0%)	-
None (n, %)	12 (19.4%)	0 (0%)	12 (85.7%)	-
Worsening (n, %)	2 (3.2%)	0 (0%)	2 (14.3%)	-
Effect on urinary incontinence				
Total positive effect (n, %)	18 (29.5%)	16 (34%)	2 (14.3%)	0.202
Substantial (n, %)	5 (8.2%)	5 (10.6%)	0 (0%)	-
Moderate (n, %)	13 (21.3%)	11 (23.4%)	2 (14.3%)	-
None (n, %)	39 (63.9%)	29 (61.7%)	10 (71.4%)	-
Worsening (n, %)	4 (6.6%)	2 (4.3%)	2 (14.3%)	-
Effect on cognition				
Total positive effect (n, %)	11 (18%)	9 (19.1%)	2 (14.3%)	1
Substantial (n, %)	6 (9.8%)	5 (10.6%)	1 (7.1%)	-
Moderate (n, %)	5 (8.2%)	4 (8.5%)	1 (7.1%)	-
None (n, %)	40 (65.6%)	33 (70.2%)	7 (50%)	-
Worsening (n, %)	10 (16.4%)	5 (10.6%)	5 (35.7%)	-

**Fig. 1:**
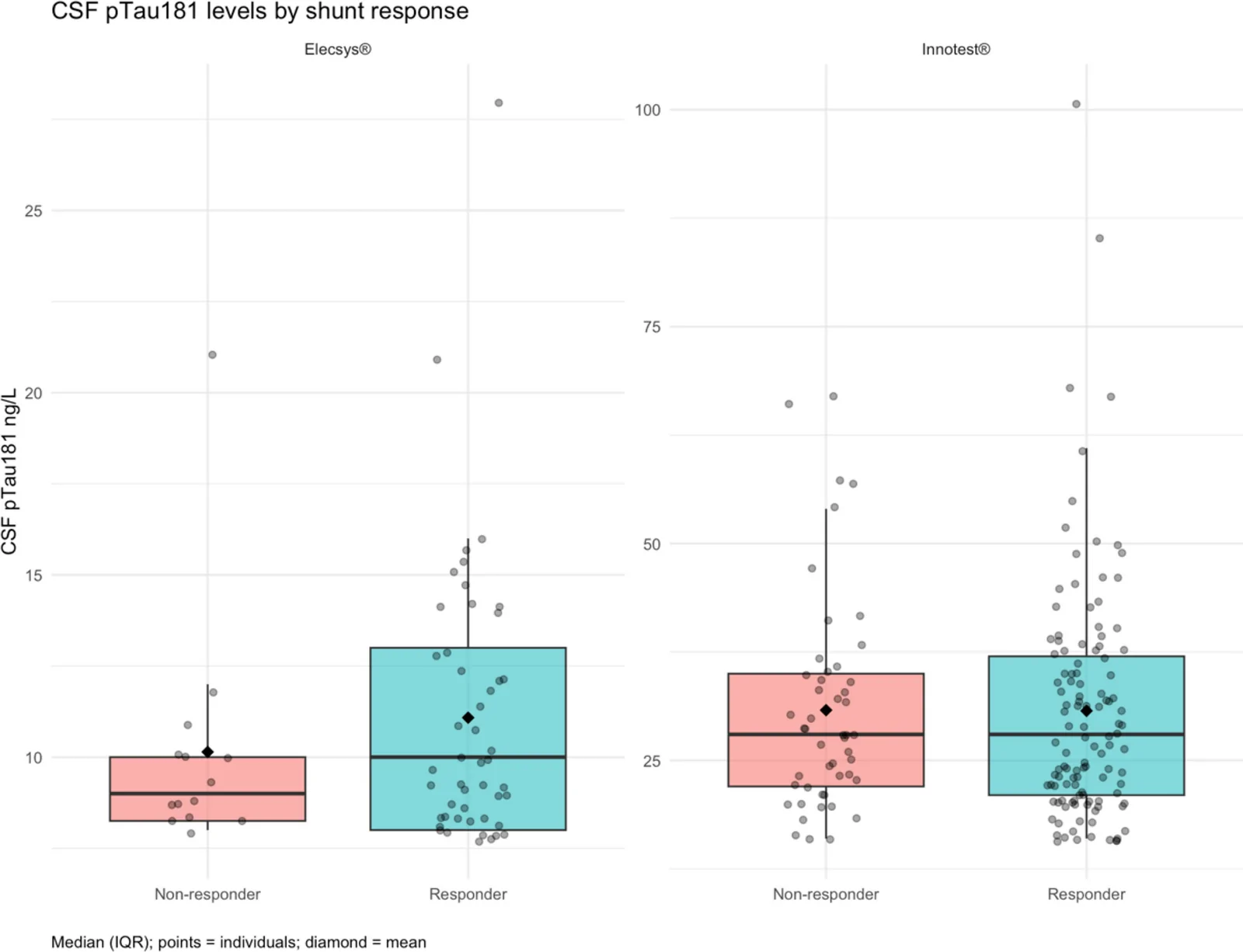
Boxplot for CSF p-tau181 levels by shunt response

Absolute CSF p-tau181 levels differed between assays, reflecting differences in analytical methods. In the Innotest^®^ cohort, mean CSF p-tau181 levels were 30.7 ± 13.8 in responders and 30.8 ± 12.4 in non-responders, with no statistically significant difference between groups (*p* = 0.829). Similarly, in the Elecsys^®^ cohort, mean CSF p-tau181 levels were 11.1 ± 3.9 in responders and 10.1 ± 3.3 in non-responders, with no statistically significant difference (*p* = 0.365) as illustrated in Fig. 1.

In both cohorts, CSF p-tau181 was not associated with shunt response in multivariable logistic regression analyses adjusted for age and sex (Innotest^®^: OR 0.997, 95% CI 0.972–1.024, *p* = 0.815; Elecsys^®^: OR 1.091, 95% CI 0.910–1.419, *p* = 0.429). When baseline gait score was included in the analyses, the observed lack of association between CSF p-tau181 and shunt response remained unchanged (Innotest^®^: OR 1.001, 95% CI 0.975–1.029, *p* = 0.956; Elecsys^®^: OR 1.062, 95% CI 0.890–1.376, *p* = 0.564).

**Fig. 2:**
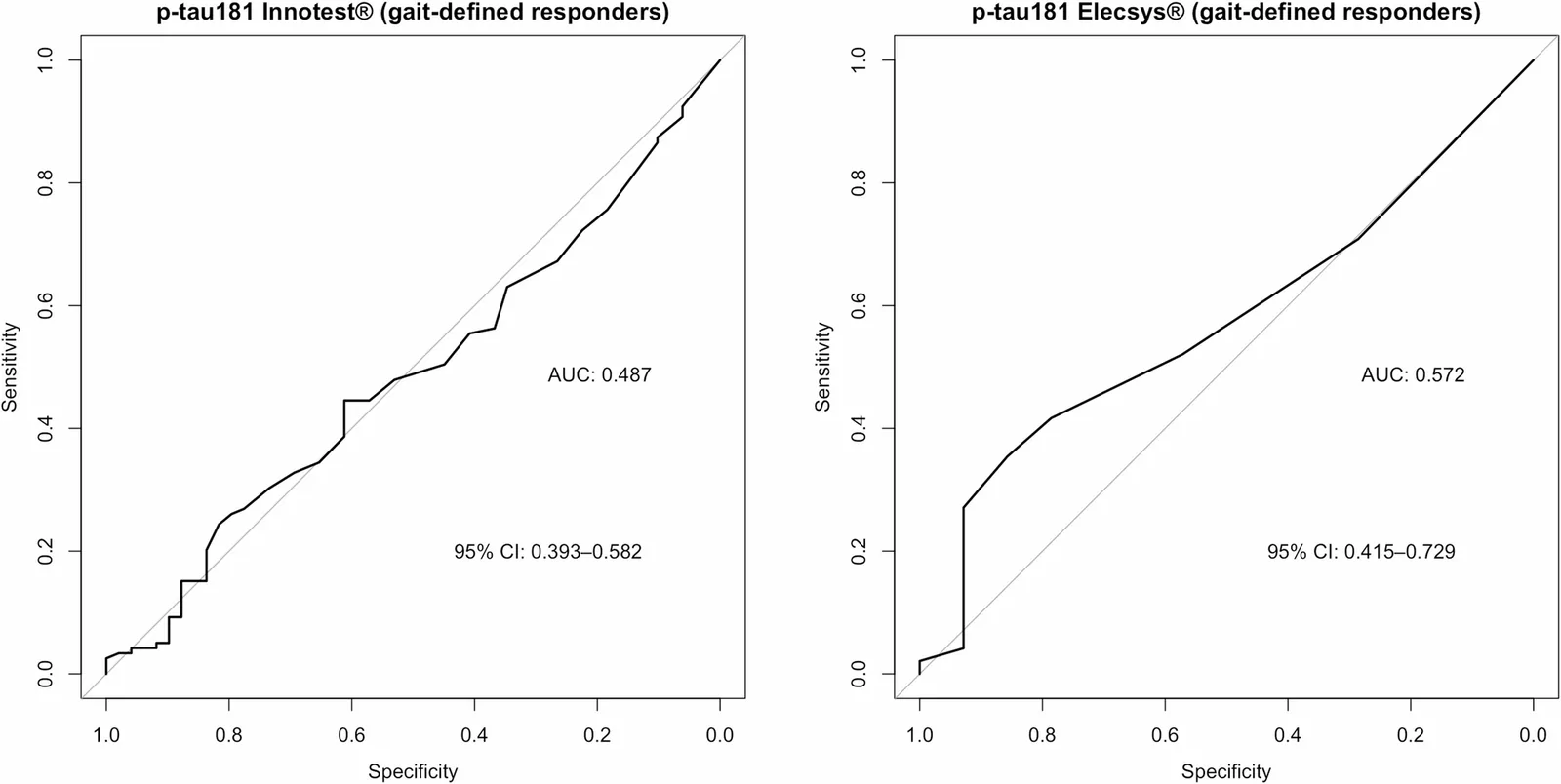
Receiver operating characteristic curves for CSF p-tau181 in predicting gait response after shunt surgery

ROC analysis demonstrated poor discriminatory performance of CSF p-tau181 for predicting shunt response. In the Innotest^®^ cohort, the AUC was 0.487 (95% CI: 0.393–0.582), while comparably limited discriminatory performance was observed in the Elecsys^®^ cohort, with an AUC of 0.572 (95% CI:0.415–0.729) (Fig. 2). Additional ROC/AUC analyses with a more stringent responder definition, defining responders as patients with substantial gait improvement, supported the primary findings with AUC values below 0.601. Similar results with poor discriminatory performance were observed when cognitive response, continence and overall clinical response were used as secondary outcome definitions. Detailed results are provided in the Supplementary Material.

Exclusion of patients with 10%, 20% and 30% highest CSF p-tau181 levels did not alter the ability of CSF p-tau181 to predict shunt response (see Supplementary Material).

When restricting the analysis to patients without neurological or musculoskeletal comorbidities, no significant difference in CSF p-tau181 was found between responders and non-responders in the Innotest^®^ cohort (see Supplementary Material). The corresponding analysis was not performed on the Elecsys^®^ cohort due to limited sample size.

## Discussion

The present study did not replicate previous findings suggesting that lower CSF p-tau181 predicts shunt responsiveness in iNPH. Contrary to our hypothesis, CSF p-tau181 was not associated with treatment response, with effect estimates close to null. This was seen for gait improvement, the primary outcome, which previously has been shown to be the most treatment-responsive symptom in iNPH [43, 44]. Likewise, CSF p-tau181 did not predict the secondary outcomes; improved cognition, continence, or overall clinical response. Although severity of baseline gait impairment was associated with shunt response, adjustment for baseline gait impairment did not alter the lack of association between CSF p-tau181 and treatment outcome.

When adjusting for concomitant AD pathology by excluding patients with the highest 10%, 20%, and 30% of CSF p-tau181 levels, the results remained unchanged. Exclusion of patients with neurological or musculoskeletal comorbidities did not alter the findings in the Innotest^®^ cohort, suggesting that such comorbidities were unlikely to explain the lack of association between CSF p-tau181 and shunt response.

One reason for p-tau181 not being able to predict shunt response could be that p-tau181 reflects underlying neurodegenerative pathology rather than the potentially reversible CSF dynamics characteristic of iNPH. Specifically, proteins are cleared from interstitial fluid via perivascular pathways and the glymphatic system [45], facilitating exchange between CSF and interstitial fluid. Impaired clearance through these pathways has been proposed to contribute to altered CSF biomarker profiles in iNPH [22]. However, CSF p-tau181 levels are not solely determined by CSF dynamics and should not be interpreted as a pure marker of impaired clearance, as their levels are also influenced by concomitant neurodegenerative pathology such as Alzheimer’s disease. Importantly, our sensitivity analyses, excluding patients with elevated p-tau181 levels did not alter the results, suggesting that coexisting neurodegenerative pathology did not explain the lack of association. Although low CSF p-tau181 to some extent may reflect altered CSF dynamics and impaired protein clearance, this does not necessarily translate into predictive value for clinical response.

Available studies have reported conflicting results on the association between p-tau181 and shunt response. Studies addressing iNPH are often limited by small cohort sizes. With 230 patients meeting the inclusion criteria, the present study represents one of the largest cohorts to date evaluating the potential predictive value of p-tau181 in iNPH. Our negative findings contrast with some earlier reports [24–30] but are consistent with several studies that did not find a predictive value of CSF p-tau181 [31–38]. Two systematic reviews have evaluated the predictive value of CSF biomarkers for shunt response in patients with iNPH [46]. A systematic review by Pfanner et al. (2018) suggested that CSF p-tau181, together with NFL, tau, Aβ42, and LRG, may represent the most promising biomarkers for predicting shunt response, although most individual studies did not find significant associations between CSF p-tau181 levels and postoperative outcomes [47]. Yet, a more recent systematic review by Thavarajasingam et al. (2022) identified one study supporting p-tau181 as a predictive factor and four studies that did not support this [46].

A clinical study not included in the review by Darrow et al. (2022) reported moderate discriminatory ability of CSF p-tau181 for gait improvement (AUC of 0.73; 95% CI: 0.48–0.95) [29]. Recently, Grønning et al. (2025) reported that CSF p-tau181 levels were not associated with shunt response in either gait or cognitive outcomes in a cohort of 113 patients [38], confirming findings from some earlier studies.

One possible explanation of these vastly negative findings may relate to the heterogeneity of the clinical presentation and underlying pathophysiology in patients with iNPH [2, 48]. Different contributing mechanisms – including altered CSF dynamics, vascular pathology, and varying degrees of concomitant neurodegenerative disease – may influence both symptom development and response to shunt surgery. Also, differences in study design, statistical methodology, outcome definitions (gait, cognition, composite clinical scores), cohort size, and duration of follow-up complicate direct comparisons across studies and may partly explain the discrepancies. Smaller cohorts may overestimate associations, and variation in outcome definitions – particularly between cognition and gait – may contribute to inconsistent findings. Although definitions of clinical improvement vary across studies, the present study did not identify predictive value of CSF p-tau181 regardless of the outcome definition applied, including gait, cognition, continence, or overall clinical response.

Taken together, these findings suggest a more fundamental limitation of CSF biomarkers as predictors of shunt response. Shunt surgery primarily targets disturbed CSF circulation, whereas biomarkers such as p-tau181 mainly reflect neurodegenerative processes. As p-tau181 is a biomarker of neurodegeneration, one might expect CSF p-tau181 levels to be lower in patients who respond favorably to shunt surgery compared with those who do not, particularly among patients showing cognitive improvement. Clinical improvement after shunt surgery may reasonably be expected to depend, at least in part, on the reversibility of symptoms related to disturbed CSF dynamics. Consequently, biomarkers reflecting neurodegeneration may not necessarily predict the degree of motor improvement following shunt surgery. This biological mismatch may partly explain why CSF p-tau181 did not demonstrate predictive value in the present study as well as in several other studies [17].

The presence of concomitant neurodegenerative pathology, such as Alzheimer’s disease, cannot be fully excluded, although we attempted to address this through sensitivity analyses. Excluding the highest 10%, 20%, and 30% CSF p-tau181 values is a pragmatic but crude approach and was not intended to diagnose AD, but rather to reduce the potential influence of patients with concomitant AD pathology who may have elevated CSF p-tau181 levels. Alternative approaches, such as using the p-tau181/Aβ42 ratio to identify likely AD pathology, have been reported with moderate discriminatory ability [49] and were therefore not employed. Further limitations of the present study include its single-center setting and retrospective design, the latter of which introduces a risk of selection bias. Patients with greater baseline gait impairment may have greater potential for measurable improvement following shunt surgery. Although additional analyses including baseline gait score did not alter the findings, the influence of baseline gait severity cannot be completely excluded, and different gait outcome measures may have yielded different results. Furthermore, the difference in follow-up time between responders and non-responders in the Elecsys^®^ cohort may have influenced the assessment of shunt response. While longer follow-up may provide greater opportunity to detect clinical improvement, treatment effects may also diminish over time [50], making the potential influence of different follow-up times difficult to estimate. Information regarding patients lost to follow-up was not available.

Strengths of the present study include the large cohort size and the consistent findings across two different CSF p-tau181 assay platforms, which support the robustness of the observed lack of predictive value, although we acknowledge the small sample size in the Elecsys^®^ cohort.

## Conclusion

Taken together, our findings suggest that CSF p-tau181 cannot be used as a predictor of shunt response in iNPH. Furthermore, adjusting for possible AD pathology did not lead to any improvement in predicting shunt responders. Although we did not find any support for p-tau181 as a predictor of shunt response, p-tau181 is effective in detecting AD pathology and we speculate that a combination of different CSF biomarkers may lead to a better predictive tool. Future research should focus on multimodal prediction models integrating other biomarkers as well as clinical and radiological markers.

## Supplementary Information

Below is the link to the electronic supplementary material.

Supplementary file1 (DOCX 3763 KB)

## Data Availability

The datasets generated and/or analysed during the current study are not publicly available due to patient confidentiality and data protection regulations. Any requests for access would be subject to institutional and regulatory approval.
