# Supplementary material for: Association between p-tau181 in cerebrospinal fluid and shunt response in patients with normal-pressure hydrocephalus
Source: Neurol Sci. 2026 Sep 7;47(10):759. doi: 10.1007/s10072-026-09357-x (PMC13547186; doi:10.1007/s10072-026-09357-x)
Supplement: Supplementary file 1 [file 10072_2026_9357_MOESM1_ESM.docx]

# Association Between p-tau181 in Cerebrospinal Fluid and Shunt Response in Patients with Normal-Pressure Hydrocephalus

## Supplementary Material

### ROC/AUC – Innotest^®^ cohort, Gait-defined responders without neurological or musculoskeletal comorbidities

### T-test – Innotest^®^ cohort, Gait-defined responders without neurological or musculoskeletal comorbidities

| **Assay** | **Biomarker** | **Non-responders mean (SD)** | **Responders mean (SD)** | **p-value** |
| --- | --- | --- | --- | --- |
| Innotest^®^ | p-tau181 | 27 (6.5) | 30.4 (14.2) | 0.231 |

### ROC/AUC – Gait-defined responders without top 10%, 20% and 30% p-tau

### ROC/AUC – Cognition defined responders

### ROC/AUC – Continence defined responders

### ROC/AUC – Overall effect-defined responders

ROC/AUC – Substantial gait improvement vs. all other outcomes

ROC/AUC – Substantial gait improvement vs. non-responders

Approval Document
